# Editorial: Common pathogenic mechanism of cerebrovascular disease and degenerative diseases

**DOI:** 10.3389/fnins.2023.1233204

**Published:** 2023-06-23

**Authors:** Wei Zheng, Yuqing Liang, Dongsheng Fan, Ji He

**Affiliations:** ^1^Department of Neurology, Peking University Third Hospital, Beijing, China; ^2^Beijing Key Laboratory of Biomarker and Translational Research in Neurodegenerative Diseases, Beijing, China; ^3^Key Laboratory for Neuroscience, National Health Commission/Ministry of Education, Peking University, Beijing, China; ^4^Peking University Health Science Center, Beijing, China; ^5^Biomedical Pioneering Innovation Center (BIOPIC), Peking University, Beijing, China

**Keywords:** cerebrovascular disease, neurodegenerative disease, pathologic mechanism, immune response, NIID

Clinically, cerebrovascular and degenerative diseases appear to be two completely different types of condition. Many notable advancements have been made in diagnosis and treatment of both diseases. However, the pathogenic mechanism of cerebrovascular disease and neurodegenerative diseases, which have complicated clinical features and various classifications, remains unclear. With the development of research, from genetic to clinical practices, there are many reports that have identified common features of these two disease families, and developing a better understanding of these factors is vital for improving diagnosis and treatment of degenerative neurological diseases. This Research Topic included inspiring opinions focusing on the pathogenic mechanism of cerebrovascular disease and degenerative diseases.

Neuronal intranuclear inclusion disease (NIID), a progressive neurodegenerative disease, is caused by GGC repeat expansion in NOTCH2NLC gene (Tian et al., 2019). NIID has the imaging characteristics of cerebrovascular disease, as well as unique pathological manifestations (Yang et al., 2022). Tai et al. used non-invasive three-dimensional arterial spin labeling for measuring cerebral blood flow. They found NIID patients exhibited decreased perfusion in the cortex but increased perfusion in the deep brain regions compared with healthy controls, showing a high-to-low gradient from the deep brain to the cortex. These findings suggested that cerebral perfusion change might act a critical role in NIID pathophysiology and could serve as a promising biomarker for monitoring NIID progression. In another study, Zhao et al. presented two cases of NIID diagnosed based on NOTCH2NLC gene testing and skin biopsy. They also reviewed all published NIID literature with positive skin biopsy and NOTCH2NLC gene results. Similarly, for brain magnetic resonance imaging, 80% of NIID patients showed high signal at corticomedullary junction on diffusion weighted imaging. These findings suggested that cerebral perfusion change may act a critical role in NIID pathophysiology and could serve as a promising biomarker for monitoring NIID progression.

Immune responses are important parts of the pathophysiology of cerebrovascular diseases, as their dynamic changes affect the development and prognosis of cerebrovascular diseases (Candelario-Jalil et al., 2022). Many studies suggested that similar immune responses play key roles in stroke and other neurodegenerative diseases (Chidambaram et al., 2022). For cerebrovascular disease, peripheral inflammation is very important in the development of stroke, acute injury cascade and pathophysiology of chronic course. For an apparent example, atherosclerosis is an inflammatory disease. In addition to the classical risk factors, peripheral immune abnormalities would also lead to an increased risk of stroke.

Cerebral edema is a common complication after stroke. Inflammatory response has been increasingly recognized recently. Gu et al. reviewed the classification (cytotoxic edema, ionic edema and vasogenic edema) and pathological characteristics of cerebral edema. The review also discussed the possible role of some factors, including aquaporin 4, microRNA, cerebral venous reflux, and cerebral ischemia/reperfusion injury, during formation of cerebral edema after ischemic stroke. This review highlighted the mechanisms of cerebral edema after ischemia stroke, providing novel therapeutic targets for prevention and treatment cerebral edema.

The pathogenesis of multiple sclerosis (MS), a kind of typical immune diseases of central nerve system, is complicated and unclear (Oh et al., 2018). Except for autoimmunity, blood-brain disruption and cerebral endothelial cells dysfunction have been realized in development of MS (Cramer et al., 2015; D'haeseleer et al., 2015). Liu et al. investigated and analyzed retinal and choroidal microvascular changes by optical coherence tomography angiography in MS patients. They found the retinal superficial vascular complex and peripapillary vessel density were reduced in eyes of MS patients, especially in patients with optic neuritis.

Similarly, accumulating evidence have demonstrated immune system participated in pathogenesis of neurodegenerative diseases, for example, amyotrophic lateral sclerosis (ALS) (McCombe et al., 2020). However, the causal relationship of dysregulated natural killer (NK) cells, as an important component of innate immunity, and the risk of ALS, remains unknown. Gong et al. performed a mendelian randomization analysis to evaluate the causal relationship between NK cells-related immune traits and ALS. The results demonstrated higher expression levels of CD16^−^CD56^+^ on NK cells and HLA-DR^+^ cells were associated with a lower risk of ALS. This work enhanced the current understanding of association of the NK-based peripheral immunity with ALS and providing insight into new therapeutic strategies target on NK cells in ALS.

Taken together, the recent research published in this Research Topic have studied pathogenesis of cerebrovascular disease and degenerative diseases from different levels and perspectives, making contributions to our understanding of neurological diseases, improving therapeutic approaches for neurological diseases. Hopefully, this Research Topic would motivate researchers to study the mechanisms of cerebrovascular disease and degenerative diseases from basic science research to clinical applications. On the other hand, articles in this Research Topic provided novel opinions regarding therapeutic approaches in neurological diseases, with more effective outcomes and fewer side effects. We look forward there would be more promising effort in the future.

## Author contributions

WZ and YL reviewed all publications and wrote the manuscript. DF and JH revised the manuscript. All authors contributed to this editorial and approved it for publication.
